# The impact of point-of-care testing for influenza A and B on patient flow and management in a medical assessment unit of a general hospital

**DOI:** 10.1186/s13104-020-04986-7

**Published:** 2020-03-10

**Authors:** S. O’Connell, C. Conlan, M. Reidy, C. Stack, A. Mulgrew, J. Baruah

**Affiliations:** 1 Pathology Department, Bon Secours Hospital, Strand Street, Tralee, Co. Kerry Ireland; 2 Shannon Applied Biotechnology Centre, Institute of Technology Tralee, Tralee, Co. Kerry Ireland; 3 Pharmacy Department, Bon Secours Hospital, Strand Street, Tralee, Co. Kerry Ireland; 4 Infection Control Department, Bon Secours Hospital, Strand Street, Tralee, Co. Kerry Ireland; 5 Consultant Respiratory Physician, Bon Secours Hospital, Strand Street, Tralee, Co. Kerry Ireland

**Keywords:** Point-of-care, Influenza, ID Now, Infection control, GeneXpert

## Abstract

**Objectives:**

Timely implementation of influenza infection control and treatment can significantly reduce the impact on Hospital resources and patient management when demand is at peak. Turnaround times of Laboratory based screening tests for the diagnosis of influenza may have an impact on the implementation of infection control measures and treatment. In this study the objectives included determining the correlation between the Abbott ID NOW point-of-care testing (POCT) instrument using the Influenza A&B2 test and the laboratory based GeneXpert Flu+RSV kit. In addition the impact of the POCT instrument on the prescription of antivirals and antibiotics was evaluated by comparing with practice when the instrument was not in place.

**Results:**

The results of the correlation study with a cohort of 54 patients revealed the Abbott ID NOW POCT has 92% sensitivity for the detection of Influenza A, while specificity was 100% for both Influenza A and B. The impact of the POCT instrument on the frequency of prescription of antivirals and amount of antibiotics consumed (33% reduction in antibiotic consumption in a cohort of 65 (2017) and 61 (2018)) was significant. In addition the average patient length of Hospital stay was significantly reduced from 5.26 days to 3.73 days.

## Introduction

The management of patient flow and bed allocation is an important element in an effective infection control programme for viral Influenza infection [1]. An effective programme requires results of screening tests to be available to make decisions in a timely manner that suit the flow of newly admitted patients through the Hospital. This is difficult with laboratory-based testing, especially at weekends and evenings where limited on-call services may only be available [2]. The nicking enzyme nucleic acid (NEAR) isothermal amplification technique enables rapid amplification in a very narrow temperature range. This eliminates the need for thermal cyclers with the high-temperature DNA denaturation cycle required for polymerase chain reaction (PCR). The low-footprint bench-top instruments can provide results in 15 min in a near-patient setting. It has been previously demonstrated that POCT leads to rapid confirmation of influenza and allows informed early decision making, reducing flu contacts and bed closures [3].

Emerging evidence suggests that less invasive samples may have comparable sensitivity to nasopharyngeal swabs or aspirates when using molecular diagnostic tests [4]. Some test kits utilize a nasal swab, which is more comfortable for the patient (e.g. ID NOW). A number of studies have been reported outlining the performance of the Alere I POCT (now known as Abbott ID NOW) NAAT versus laboratory based methods such as thermocycler rtPCR and viral culture for Influenza testing [5–16]. These studies have reported influenza A sensitivity in the range of 66–100% and specificity of 55–100%, while for influenza B sensitivity ranged from 45 to 100% and specificity 54–100%. The studies comparing ID NOW with the GeneXpert have previously used nasopharyngeal swabs in viral transport medium (VTM) [6–8]. Sensitivities were reported to be lower when VTM specimens were used instead of direct nasal swabs. The assay also had lower sensitivity with samples determined to have low Flu A titers as determined by higher Ct values in rtPCR [5].

In order to better assist with patient flow and bed management in winter, we prospectively assessed the utility of the POCT ID NOW Influenza A&B2 assay. The test was compared with the GeneXpert platform in a laboratory setting. In addition the impact on antibiotic and antiviral prescriptions was compared to determine the influence of the POCT service on these elements of patient management in 2 consecutive flu seasons (2017 pre-implementation and 2018 post-implementation).

## Main text

### Methods

#### Suspected influenza infection control pathway/algorithm

The infection control algorithm for suspected influenza implemented during the pre and post POCT testing period of the study are outlined in the supplementary information. In the context of the COVID-19 outbreak the Hospital infection control algorithm for suspected COVID-19 can be accessed at [17], with entry to the suspected influenza pathway occurring on being ruled out of the COVID-19 pathway.

#### Specimen Collection

Matched patient specimens were collected in the medical assessment unit of Bon Secours Hospital, Tralee, Co. Kerry, Ireland, which met the Hospital’s clinical algorithm for suspected influenza between October 2018 and April 2019. A mid turbinate nasal swab (Puritan sterile foam tipped applicator, Ref 25-15061PF) tested within 2 h of collection was the preferred specimen for the ID NOW POCT device. Nasal and throat specimens for analysis on the GeneXpert were collected using Cepheid Xpert^®^ Nasopharyngeal Sample Collection kit for Viruses (Ref SWAB/B-100) with viral transport medium (VTM). Specimens for testing with the GeneXpert Flu+RSV assay were typically stored at − 20 ℃ for up to 24 h during routine days and up to 72 h at weekends prior to transport to the referral laboratory for testing.

#### Clinical implementation and training

The POCT Co-Ordinator, MAU nursing staff and medical Senior House Officers (SHO’s) were trained by the manufacturer on how to use the ID NOW instrument. Patients who had a positive Influenza result were flagged for standard droplet precautions and isolation by the infection control team.

#### Specimen analysis

Patient specimens were analysed on both test systems as per manufacturer’s instructions. Each batch of reagents used in the validation study were quality controlled on receipt. Specimens were tested with the Abbott ID NOW A&B2 assay within 2 h of collection. Analysis of the patient swabs using the GeneXpert platform by the referral laboratory was carried out by mixing an equal volume aliquot of VTM from both the nasal and throat specimens in the test to give an aggregate sample. The GeneXpert method provided a positive result if either the nasal or the throat swab was positive in the pooled sample.

#### Test data collection

The collection details and results for specimens analysed using the ID NOW were manually recorded on an influenza specimen analysis report compiled by the operator which was subsequently filed in the patients medical chart. Specimens for analysis using the GeneXpert were sent to the pathology department. The specimens were registered on the Laboratory Information System (iSOFT) and transferred by courier to the referral laboratory.

#### Collection of data on the management of influenza-positive patients

Data was gathered from the Hospital LIS and Patient Information Management System (PIMS, iSoft) which were retrospectively interrogated using SAP Business Objects (BI Platform 4.1 Version: 14.1.6.1785). Data on all patients who had specimens collected for influenza testing from September 2017 to April 2018 represented the pre POCT testing data set. Data on all patients who had a point-of-care influenza test from September 2018 to April 2019 represented the post POCT implementation data set.

#### Calculation of defined daily dosing of antibiotics

The World Health Organisation describes the Defined Daily Dose (DDD) as the assumed average maintenance dose per day for a medicine used for its main indication in adults [18]. The selection criteria included only adult patients who had a confirmed diagnosis of Influenza Type A or B. A multidisciplinary team reviewed medical notes and prescriptions to determine generic antibiotic name, dose, number of doses and cumulative dose of antibiotic administered during inpatient admission for the respective cohort periods. In accordance with WHO Collaborating Centre for Drug Statistics Methodology, antibiotic consumption data were collated and analysed using the 2018 reference values available through the Anatomical Therapeutic Chemical (ATC) classification system [19]. DDDs were calculated for each antimicrobial consumed for every patient suitable for cohort entry.

#### Statistical analysis

Suitable statistical analysis was performed using the Microsoft Excel add on XLSTAT. A p-value of < 0.05 was considered to be statistically significant. The Chi squared test was used for categorical data.

### Results

#### ID NOW influenza A&B2 test validation

A total of 54 patient specimens were tested during the validation phase. The results of the influenza A and B correlation are presented in Additional file 1: Tables S1 and S2, respectively. There was one discrepant result between the 2 instruments for influenza A. Repeat analysis of the nasal and throat specimen separately on the GeneXpert resulted in a Ct value of 36.7 for the nasal swab while the throat swab was negative. There were no influenza B positive patient specimens obtained during the validation period. External quality control scheme samples and internal quality control samples positive for influenza B were tested by both systems with 100% agreement.

The performance characteristics from the validation study are presented in Additional file 1: Table S3. The ID NOW was found to have a sensitivity for Influenza A of 92% and a specificity of 100%, while for Influenza B, 100% was obtained for both sensitivity and specificity.

#### Age and gender profile of patients screened for influenza

The age and gender profile of the patient cohort screened for Influenza during 2017 and 2018 is outlined in Additional file 1:Table S4. There were more patients screened during the 2017 season than 2018, with more females than males screened in both seasons. The average age of patients screened was significantly younger in 2018 (p-value = < 0.05).

#### Prevalence and management of influenza-positive patients

The results of influenza screening were retrospectively collated for the 2017 and 2018 seasons. It is evident from the results (Table 1) that Influenza B was the dominant strain of Influenza during the 2017 season with influenza A dominant in 2018. The incidence of influenza-positive specimens was 22.3% and 22.4% in 2017 and 2018, respectively. The number of patients receiving Oseltamivir increased from 23 in 2017 to 51 in 2018. In addition the number of patients receiving Oseltamivir as a result of a positive Influenza screening test also increased from 18 to 42. The average defined daily dose (DDD) of antibiotic for the cohort of patients was found to reduce in 2018 (30.7%). The implementation of infection control measures such as patient isolation and cohorting of influenza-positive patients increased from 52% in 2017 to 70% in 2018. A statistically significant (p = 0.003) reduction in the average bed stay was observed from an average of 5.26 bed nights in 2017 to 3.73 in 2018.

**Table 1:** Profile of Influenza prevalence and management in two consecutive seasons without (2017/18) and with POCT Influenza testing (2018/19)

Parameter	2017			2018		
No. patients screened for influenza A and B	292			272		
No. of influenza-positive patients	A	B	Total	A	B	Total
	23	42	65	60	1	61
No. of patients receiving Oseltamivir	23 (8%)			51 (19%)		
No. of influenza-positive patients receiving Oseltamivir	18 (28%)			42 (69%)		
Defined daily dosing (DDD) for antibiotics	191.6			132.7		
No. of patients isolated/cohorted on admission	34 (52%)			43 (70%)		
Average influenza-positive patient length of stay (bed nights)	5.26 ± 3.4			3.73 ± 2.1*		

*p = 0.003

#### Profile of antibiotic prescribing

A sample of 22, adult (≥ 18 years) influenza-positive patient charts, from 2017 to 2018 seasons, were reviewed to evprofile of the patient cohortaluate the profile of defined daily doses of antibiotics administered (Table 2).

**Table 2:** Defined daily dosing profile of antibiotics prescribed to influenza-positive patients during the 2017 and 2018 seasons

Antibiotic	2017	2018
Amoxicillin	3.0	0.0
Azithromycin	8.3	2.3
Cefixime	4.5	0.0
Cefotaxime	14.5	10.8
Ceftriaxone	5.0	3.5
Clarithromycin	67.0	63.0^*^
Co-amoxiclav	43.9	41.7
Co-trimoxazole	12.5	0.0^*^
Gentamicin	11.7	0.0^*^
Levofloxacin	0.0	4.0^*^
Metronidazole	5.0	1.0
Moxifloxacin	5.0	0.0
Piperacillin/Tazobactam	11.3	6.4
Total	191.6	132.7

*p < 0.05, Chi Sq = 39.687, Chi Sq Critical value = 21.026, n = 22

It is evident from the results that there was a significant reduction in the number of antibiotic doses prescribed in 2017 versus 2018 (p < 0.001). There were a total of 11 different antibiotics used in 2017, while this was found to reduce to 7 in 2018 with a concurrent reduction of 58.9 in the total number of doses (30.7%) consumed in this patient cohort.

## Discussion

The data from this study indicates a compelling positive correlation between POCT influenza diagnostics, early initiation of targeted antiviral therapy (where indicated) and a notable reduction in antimicrobial consumption in the study cohort.

In this study the performance of the ID NOW Flu A&B2 versus the GeneXpert is similar to that previously reported [16, 20]. The one discordant specimen was found to have a low viral load in the nasal specimen as determined by the threshold cycle (Ct) values obtained from the GeneXpert. Analysis of the same samples on both systems ruled out any swab sampling associated bias. It is likely that the differences between the 2 methods are associated with the Ct cut-off values which are typically optimised based on the correlation with viral culture and TCID_50_/ml (Tissue culture infecting dose) values for specific viral strains. Consensus on a clinically relevant Ct cut-off for a positive specimen would be useful for assessing NAAT POCT devices.

In the 2017/2018 season 4713 people in Ireland with influenza were hospitalised and 191 people needed admission to critical care units. A total of 255 people with notified influenza died [21]. The incidence of Influenza in both seasons 2017 and 2018 in our study was 22% of those patients screened. The amount of patients screened for influenza relative to admissions was also similar for both seasons. The average age of patients screened for influenza dropped significantly in 2018 when compared to 2017. The uptake of influenza vaccination in Ireland during the period September-December 2018 in those aged 65 years and older was 56.0% (2017/18 season 55.1%). Variation in vaccination coverage was observed between age groups, with the highest uptake (60.3%) in those aged 75 years or older [22]. These vaccination statistics may partly explain the reduction in the average age of patients being screened for influenza infection.

The results obtained suggest that repeatability of a 33% reduction in antibiotic consumption and overall study reproducibility can be attained and supported by the implementation of a robust antimicrobial stewardship programme [23].

The introduction of the Abbott ID NOW Influenza A&B2 test at the point-of-care in the Hospital medical assessment unit allowed an identified issue with delayed diagnostic test results in the suspected Influenza care pathway to be addressed. The point-of-care test allowed implementation of a more effective and efficient care pathway which was associated with a reduced hospital stay and a reduction in antibiotic consumption.

### Limitations

Further studies in larger patient populations would be useful to demonstrate the reproducibility of this study which has the potential to lead to significant cost savings and better bed management during peak influenza season. The effects of variable individual prescriber preference for antibiotics, laboratory cultures and sensitivities and late patient presentation to hospital on statistical significance of antibiotic usage patterns and variance between cohorts should be noted.

## Supplementary information

**Additional file 1: Tables S1–S3.** The correlation of the ID NOW and GeneXpert. **Table S4.** The demographic details of the patient cohort studied.** Figures S1, S2** Outlining the suspected Influenza care pathway implemented during the study period.

## Data Availability

Anonymised and codified datasets are available from the corresponding author on reasonable request.

## Abbreviations

POCT: Point-of-care testing
NAAT: Nucleic acid amplification tests
NEAR: Nicking enzyme nucleic acid restriction
VTM: Viral transport medium
PCR: Polymerase chain reaction
RT: Reverse transcriptase
MAU: Medical assessment unit
