# Supplementary material for: The impact of point-of-care testing for influenza A and B on patient flow and management in a medical assessment unit of a general hospital
Source: BMC Res Notes. 2020 Mar 10;13:143. doi: 10.1186/s13104-020-04986-7 (PMC7063764; doi:10.1186/s13104-020-04986-7)
Supplement: Supplementary file 1 — Additional file 1: Tables S1–S3. The correlation of the ID NOW and GeneXpert. Table S4. The demographic details of the patient cohort studied. Figures S1, S2 Outlining the suspected Influenza care pathway implemented during the study period. [file 13104_2020_4986_MOESM1_ESM.docx]

**Additional file 1**

**The Impact of Point of Care Testing for Influenza A and B on Patient Flow and Management in a Medical Assessment Unit of a General Hospital.**

O’Connell, S.^1,2*^, Conlan, C.^3^, Reidy, M.^4^, Stack, C.^3^, Mulgrew, A.^5^, Baruah, J.^1^

1. Pathology Department, Bon Secours Hospital, Strand Street, Tralee, Co. Kerry, Ireland.

2. Shannon Applied Biotechnology Centre, Institute of Technology Tralee, Co. Kerry, Ireland.

3. Pharmacy Department, Bon Secours Hospital, Strand Street, Tralee, Co. Kerry, Ireland.

4. Infection Control Department, Bon Secours Hospital, Strand Street, Tralee, Co. Kerry, Ireland.

5. Consultant Respiratory Physician, Bon Secours Hospital, Strand Street, Tralee, Co. Kerry, Ireland.

***Corresponding Author: sjpoconnell@bonsecours.ie**

**Table S1:** Correlation Between ID NOW Influenza A&B2 Test for Influenza A and the Laboratory based GeneXpert Flu + RSV.

|  | | **GeneXpert** | | | | | |
| --- | --- | --- | --- | --- | --- | --- | --- |
|  |  | **Positive Inf A** | | **Negative Inf A** | | | **Total** |
| **ID NOW** | **Positive Inf A** | | 12 | | | 0 | **12** |
|  | **Negative Inf A** | | 1 | | | 41 | **42** |
| **Total** | | | **13** | | **41** | | **54** |

**Table S2**: Correlation Between Alere I POCT Influenza A&B2 Test for Influenza B and the Laboratory based GeneXpert Flu + RSV.

|  | | **GeneXpert** | |  |
| --- | --- | --- | --- | --- |
|  | | **Positive Inf B** | **Negative Inf B** | **Total** |
| **ID NOW** | **Positive Inf B** | 0 | 0 | **0** |
|  | **Negative Inf B** | 0 | 54 | **54** |
| **Total** | | **0** | **54** | **54** |

**Table S3:** Summary Statistics for ID NOW Influenza A&B2 comparison with GeneXpert Flu+RSV

| **Statistical Measure** | **Flu A** | **Flu B** |
| --- | --- | --- |
| **Negative predictive value** | 98% | 100% |
| **Positive predictive value** | 100% | 100% |
| **Sensitivity** | 92% | 100% |
| **Specificity** | 100% | 100% |

**Table S4:** Summary of the Gender and Age Profile of the Patients Screened for Influenza during the 2017 and 2018 seasons

| **Parameter** | | **2017** | **2018** |
| --- | --- | --- | --- |
| **No. of Patients Screened** | **Male** | 122 | 127 |
|  | **Female** | 170 | 145 |
|  | **Total** | 292 | 272 |
| **Average Age** | | 63.4 | 56.1* |
| **Median** | | 65.2 | 60.5 |
| **Max** | | 92.2 | 90.0 |
| **Min** | | 7.3 | 0.8 |

^*^ - P value < 0.05,


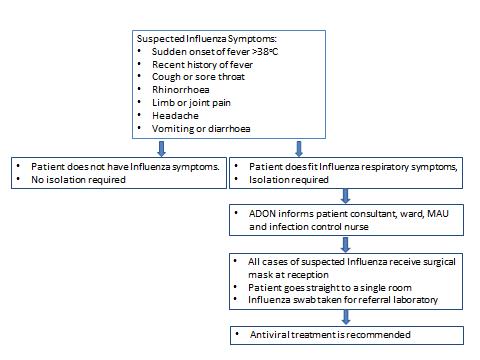


**Figure S1:** Algorithm for screening patients for influenza like symptoms without point of care testing for influenza.


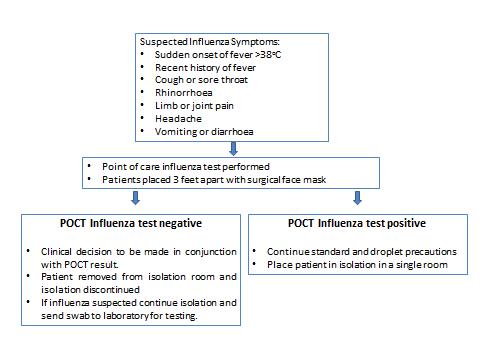


**Figure S2** Algorithm for screening patients for influenza like symptoms with the inclusion of point of care Influenza testing in the patient pathway.
